# Parental autoimmune and autoinflammatory disorders as multiple risk factors for common neurodevelopmental disorders in offspring: a systematic review and meta-analysis

**DOI:** 10.1038/s41398-022-01843-y

**Published:** 2022-03-18

**Authors:** Pierre Ellul, Eric Acquaviva, Hugo Peyre, Michelle Rosenzwajg, Pierre Gressens, David Klatzmann, Richard Delorme

**Affiliations:** 1 Child and Adolescent Psychiatry Department, Robert Debre Hospital, APHP, Paris, France; 2 Immunology-Immunopathology-Immunotherapy (i3), UMRS 959, INSERM, Paris, France; 3 NeuroDiderot, Paris University, INSERM, Paris, France; 4 University hospital department Inflammation-Immunopathology-Biotherapy (i2B), Pitié-Salpêtrière Hospital, APHP, Paris, France; 5 Human Genetics and Cognitive Functions, Institut Pasteur, Paris, France

**Keywords:** Autism spectrum disorders, ADHD

## Abstract

Epidemiological studies have raised concerns about the risk of neurodevelopmental disorders (NDD) in children of patients with autoimmune or inflammatory disorders (AID). The pathophysiological pathways underlying this association are still unknown and little is known about the specific and distinct risk of each AID. To explore these questions, we investigated the association between the occurrences of several NDD in the offspring of mothers or fathers with different IDA. We conducted a meta-analysis—*PROSPERO* (CRD42020159250)—examining the risk of NDD in the offspring of mothers or fathers with AID. We performed specific analyses separately in fathers or mothers of NDD patients as well as subgroup analyses for each NDD and AID. We searched MEDLINE, Embase, PsycINFO, Cochrane Central Register of Controlled Trials, and Web of Science Core Collection published until December 2021. From an initial pool of 2074 potentially relevant references, 14 studies were included, involving more than 1,400,000 AID and 10,000,000 control parents, 180,000 children with NDD and more than 14,000,000 control children. We found AID in mothers (Adjusted OR 1.27 [95% CI 1.03; 1.57] *p* = 0.02, [I^2^ = 65%, Tau^2^ = 0.03 *p* = 0.01] and adjusted OR 1.31 [95% CI 1.11; 1.55] *p* = 0.001, [I^2^ = 93%, Tau^2^ = 0.13 *p* = 0.001] and, although in a lesser extent, in fathers (adjusted OR 1.18 [95% CI 1.07; 1.30] *p* = 0.01, [I^2^ = 15.5%, Tau^2^ = 0.002 *p* = 0.47]) and adjusted OR 1.14 [95% CI 1.10; 1.17] *p* < 0.0001, [I^2^ = 0%, Tau^2^ = 0 *p* = 0.29]) to be associated with ASD and ADHD in the offspring. This difference in the strength of the association was found in the AID-specific analyses, suggesting that AID increase the risk of NDD by a shared mechanism but that a specific maternal route appears to represent an additional excess risk. Inflammatory bowel disease were not associated with an additional risk (neither in fathers nor in mothers) of NDD in offspring. Our results suggest that complex and multiple AID-specific pathophysiological mechanisms may underlie the association of AID and NDD in offspring. Further, comprehensive studies of the different AID and NDD are needed to draw definitive conclusions about the pathophysiological links between parental AID and NDD in children.

## Introduction

Neurodevelopmental disorders (NDD) are a group of neuropsychiatric conditions that occur in children at an early stage of development and affect more than 10% of children [1]. Based on DSM-5, NDD gather autism spectrum disorders (ASD), attention deficit/hyperactivity disorder (ADHD), developmental coordination disorder, developmental language disorder, dyscalculia, dyslexia, intellectual disability (ID), and tic disorders [2]. Their determinants result from close entanglements between genes and environment [3]. Environmental factors were identified as key players in the physiopathology of NDD [4–7]. Among them, immune-mediated events could play an important role in the etiology of NDD. For example, maternal fever during pregnancy increases the risk of NDD in the offspring [8–10]. In animal studies, this association is mediated by the direct action of the innate immune system, inducing a disruption in the brain development [11, 12]. In humans, similar maternal cytokines during pregnancy—called maternal immune activation (MIA)—affect the fetal brain development, its connectivity, and functions [13].

Autoimmune and autoinflammatory disorders (AID) are characterized by self-reactive immune system activation, which results in the synthesis of either organ-specific or systemic auto-antibodies and also the secretion of various cytokines leading to tissue damages [14]. AID represent a group of more than 100 distinct diseases affecting altogether 3–5% of the general population [14].

Several studies have highlighted the possibility of an association between AID in the family or in the mother alone and certain NDD (mainly ASD) in their children, suggesting that some of the causal factors of AID may also be involved in NDD [15–18]. No previous systematic reviews/meta-analyses have addressed this association (i) considering all the different NDD or AID, and (ii) by comparing separately fathers and mothers with or without AID. Indeed, it is not known whether AID affect fetal neurodevelopment through direct action, for example via the maternal immune system during pregnancy (or MIA), or through other pathways, such as a common genetic or environmental background. By considering fathers and mothers separately, our meta-analysis could provide epidemiological arguments in favor of one of these hypotheses.

## Methods

We performed a systematic review and meta-analysis following the PRISMA recommendations [19].

### Search strategy

We searched MEDLINE (1946 to December 2021), EMBASE (1974 to December 2021), PsycINFO (1806 to December 2021), Cochrane Central Register of Controlled Trials (CENTRAL; from inception to December 2021), and Web of Science Core Collection (1900 to December 2021) without any restrictions on language, ethnic origins of the participants, date, or article type. Search terms were reported in Supplementary Data. We have also explored the references in studies we included for any potential pertinent study not detected by the initial search strategy.

### Study selection

Studies were included if they met the following criteria: (1) explore the risk of developing at least one NDD in the offspring (according to DSM-5 definition); (2) examine the impact of one or more AID in the parents (mothers or fathers) on the NDD associated risk in their offspring (AID that were selected a priori by the American Autoimmune and Related Diseases Association and members of the Eurofever Project [20, 21]); (3) include a control group of healthy parents without a personal history of AID. Studies with a control group of individuals with a psychiatric disorder were not eligible. Exclusion criteria for the selection of the studies were: (1) assess only symptoms but not a full diagnosis of NDD; (2) diagnosis of AID using biological markers only; (3) Data not available on AID status in both mothers and fathers or NDD in offspring. Two researchers (PE and EA) independently screened title or abstract potentially pertinent and excluded those clearly not relevant. Discrepancies were resolved by a consensus. If necessary, a senior researcher (RD) acted as an arbitrator. The full-text version of the selected articles were then assessed for eligibility by the two researchers, independently. Discrepancies were resolved as describe previously. When required, corresponding authors were contacted to clarify study eligibility. The protocol for the present systematic review/meta-analysis was registered on the international Prospective Register of Systematic Reviews *PROSPERO* (protocol number: CRD42020159250). Any deviation of the published protocol is reported in the Supplementary Materials. PRISMA checklist is included in the Supplementary Data.

### Data extraction, outcomes, and evaluation

PE and EA independently extracted the data, which was cross-checked to ensure its accuracy. Variables extracted were (i) author names; (ii) year of publication; (iii) country in which the study was conducted (iv) main demographic and clinical characteristics of the population studied (age, sex ratio, number of parents, and offspring in each condition); (v) AID or NDD considered in the study (with diagnosis criteria used); (vi) adjusted OR and confidence interval (CI) (or crude odd ratio if adjusted are not available), with the adjusting factors used.

Study quality was estimated by using a modified version of the Newcastle Ottawa Scale (NOS) [22, 23]. Briefly, the NOS provided assessment criteria for case-control, cross-sectional, and cohort studies. Three methodological domains were assessed: selection criteria; comparability; measurement of outcome/exposure. Scoring criteria were amended such that the maximum score available for each study was eight. Studies were considered as high quality if the NOS score was strictly over four.

The main outcome measures were the effect size for each NDD in children assessed in mothers and fathers with and without AID. Preplanned secondary outcome measures were: (i) In case of positivity of the main outcome, the effect size based on cross-stratification between subtypes of AID in the parents and subtypes of NDD in the offspring; (ii) A sensitivity analysis by grouping cohort and case-control studies separately; (iii) A sensitivity analysis with only good quality controls according to NOS.

### Analysis

We used a random-effects meta-analysis model. Heterogeneity was assessed using the I^2^ statistic and Tau^2^. Here, we considered that a value of I^2^ > 75% represented substantial heterogeneity between studies [24]. In case of heterogeneity and if the test conditions were met, publication bias were analyzed with both contour-enhanced funnel plot and Egger’s test [25, 26]. We then checked the effect of outliers using “metainf”. Statistical analysis was performed using the R package “meta” for meta-analysis of unadjusted OR and “metaphor” for adjusted OR (log-transformed). Analysis were only performed when three or more studies were available. In the main analyses, as some studies had multiple outcomes, to limit effect size dependencies we combined the groups to create a single pairwise comparison per study [27]. Random-effects meta-regression analysis were done to quantify the association on quality scores. These analysis were performed using the function “metareg” of R package “meta” [28].

Briefly, we have first analyzed the associations between each NDD with pooled AID in fathers or mothers for unadjusted and adjusted OR. In order to ensure the validity of our results, we have carried out sensitivity analysis (i) selectively according to the type of study, (ii) only in studies considered to be of good quality.

## Results

**Fig. 1:**
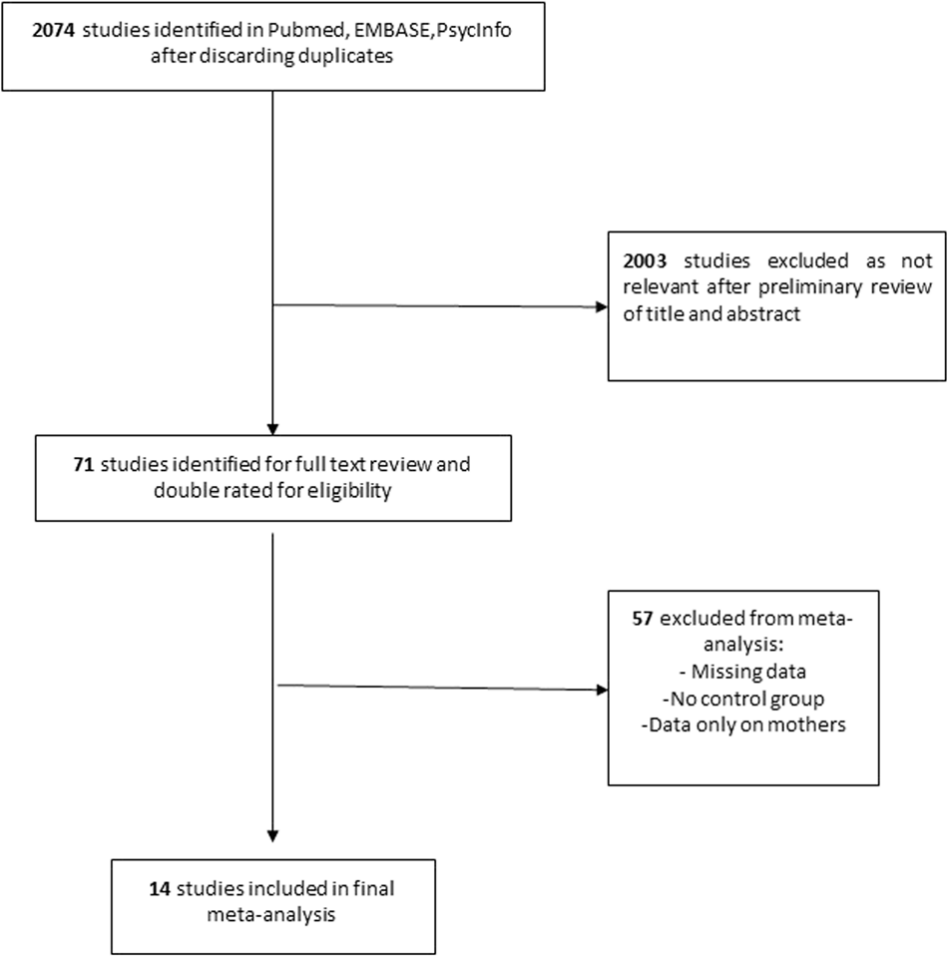
Flowchart of the selection process for studies included.

From 2074 potentially relevant references, we included 14 studies [29–42]. Figure 1 reported the flowchart detailing the screening process. Studies gathered: (i) 845,411 mothers with AID; (ii) 4,984,965 mothers from the general population as control individuals; (iii) 601,148 fathers with AID diseases; (iv) 4,992,854 fathers from the general population; (v) 182,927 children with NDD; and (vi) 14,168,474 children as control. Descriptions of the studies included in the meta-analysis are reported in Table 1 for retrospective studies and Table 2 for prospective studies. Studies included were considered as being of good quality: NOS = 5.8 ± 1.4 and 6.7 ± 0.4. We observed no significant association between quality of studies and their results in meta-regression analysis neither in mothers or fathers [log(OR) = −0.08 ± 0.1, *p* = 0.4; and log(OR) = −0.24 ± 0.22, *p* = 0.28; respectively].

**Table 1:** Descriptive data for cross-sectional studies included in the meta-analysis.

Author	Year	Country	Neurodevelopemental disease	Autoimmune disease	N of NDD	Age	Sd	Sex ratio	N of NDD with parental AID	N unexposed	Age	Sd	Sex ratio	N of controls with parental AID
Comi	1999	USA	ASD	AID	61	9.8	NR	56/5	21	46	8.7	NR	40/6	4
Sweeten	2003	USA	PDD	AID	101	10.2	4.2	83/18	31	101	6.5	4	60/41	12
Mouridsen	2007	Denmark	ASD	UC, RA, T1D, Crohn, ITP, Reiter’s	111	5.4	2.5	0.74	25	330	5.4	2.5	0.82	27
Atladottir	2009	Denmark	ASD	T1D, Connectivite tissue disease, RA, Celiac, Crohn, UC, MS, Psoriasis	3325	NR	NR	0.84	NR	689,196	NR	NR	NR	NR
Atladottir	2009	Denmark	IA	T1D, UC, Connectivite tissue disease, Psoriasis	1089	NR	NR	893/196	NR	689,196	NR	NR	NR	NR
Keil	2010	Sweden	ASD	T1D, IBD, Psoriasis, ITP,SLE, myasthenia gravis, Rheumatic fever	1237	1–10	NR	0.77	67	30,925	1–10	NR	0.76	986
Nielsen	2016	Denmark	ADHD	SLE, UC, Crohn, Psoriasis, RA, JA, AS, Addison, Celiac, Pernicious Anemia, ITP, MS, Idiopathic Polyneuritis, Iridocylitis, Autoimmune hepatitis, Alopecia Areata, Vitiligo, Polymyalgia Rheumatica, myasthenia gravis, Sclerodermia, Sjorgren	23,645 + 880	5–12	NR	NR	1010	960,035 + 982,800	5–12	NR	NR	30,843 (+10,621)
Mataix-Cols	2017	Sweden	TS	AID	7083	NR	NR	NR	1360	7,409,570	NR	NR	NR	1,313,209
Croen	2018	USA	ASD	Autoimmune hepatitis, Celiac, Crohn, Dermatitis herpetiformis, Psoriasis, Hemolytic anemia, RA, SLE, Sjorgren, Thrombocytopenia, T1D, UC	663	2–5	NR	0.82	322	915	2–5	NR	0.54	321
Croen	2018	USA	DD	Autoimmune hepatitis, celiac, Dermatisis Herpetiformis Psoriasis, Hemolytic anemia, MS, Optic neuritis RA, SLE, Sjogren, Thrombocytopenia, T1D, UC	984	2–5	NR	66/33	380	915	2–5	NR	0.54	322
Spann	2019	Finland	ASD	AID	4600	NR	NR	NR	1479	18,058	NR	NR	NR	5017
Hegvik	2021	Sweden	ADHD	AS, Celiac, Crohn, Grave’s disease, MS, Psoriasis, Hashimoto, RA, Sarcoidosis, Sjorgren, SLE, T1D, UC	118,927	NR	NR	76,113/42,814	5577	113,350	NR	NR	NR	NR

Note that for the studies of Altadottir and Croen, we have deliberately shown two different lines because they each studied two neurodevelopmental outcomes.

*AID* autoimmune or inflammatory disorders, *NDD* neurodevelopmental disorders, *T1D* type 1 Diabetes, *RA* rheumatoid arthritis, *IBD* inflammatory bowel disease, *UC* ulcerative colitis, *MS* multiple sclerosis, *SLE* systemic lupus erythematosus, *JA* juvenile arthritis, *AS* ankylosing spondylitis, *ADHD* attention deficit/hyperactivity disorders, *ASD* autism spectrum disorders, *PDD* pervasive developmental disorder, *IA* infantile autism, *TS* Tourette syndrome, *DD* developmental disorders.

**Table 2:** Descriptive data for cohort studies included in the meta-analysis.

Author	Year	Country	AID	NDD	N of parents with AID	N of parents with AID and offspring with NDD	N of parents without AID	N of parents without AID and offspring with NDD
Ji	2018	Sweden	T1D	ADHD	6700 + 15,615	NR	993,442 + 1,380,829	NR
Rom	2018	Denmark	RA	ASD	15,615 + 13,556	84	1,380,829 + 1,904,167	18,116
Andersen	2014	Denmark	IBD	ASD	6330 + 6700	62	1,911,393 + 99,442	16,050
Lee	2021	Taiwan	Sjogren, SLE, RA, Systemic sclerosis, Idiopathic inflammatory myositis, T1D, MS, Myasthenia gravis, Psoriasis, IBD, Vasculitis, AS, Behçet	ADHD	1990	117 + 61	706,527	28,092 + 28,148
Lee	2021	Taiwan	Sjogren, SLE, RA, Systemic sclerosis, Idiopathic inflammatory myositis, T1D, MS, Myasthenia gravis, Psoriasis, IBD, Vasculitis, AS, Behçet	ASD	1327	13 + 10	707,190	4493 + 4496

Note that for Lee, we have deliberately shown two different lines because they studied two neurodevelopmental outcomes.

*AID* autoimmune or inflammatory disorders, *NDD* neurodevelopmental disorders, *T1D* type 1 diabetes, *RA* rheumatoid arthritis, *IBD* inflammatory bowel disease, *ADHD* attention deficit/hyperactivity disorders, *ASD* autism spectrum disorders.

### ASD in the offspring and AID in parents

**Fig. 2:**
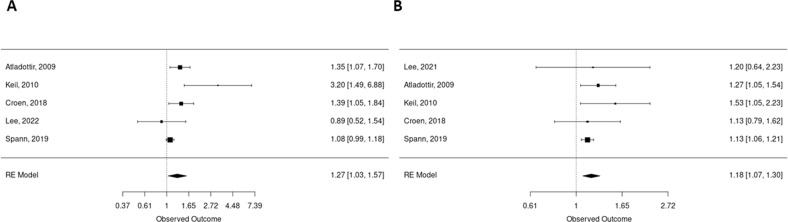
Forest plot showing the meta-analysis results of the association between AID in parents and ASD in the offspring (adjusted). **A** Mothers **B** Fathers. Each square represents individual study effect. Its size represents the study weight in the overall analysis. The black lines on either side of the squares represent the confidence intervals. The diamond at the bottom represents the summary effect with the outer edges representing the confidence intervals. Square or diamond on the right of the central bar (i.e., superior to 1) represents a positive association between maternal AID and ASD in the offspring. To be significant, the confidence interval lines must not cross 1.

We found a positive association between AID in mothers and the risk of ASD in the offspring in unadjusted analysis (six studies) (OR 1.41 [95% CI 1.09; 1.83] *p* = 0.37, [I^2^ = 69%, Tau^2^ = 0.05 *p* < 0.01]) (Supplementary Fig. 1A) and adjusted analysis (five studies) (OR 1.27 [95% CI 1.03; 1.57] *p* = 0.02, [I^2^ = 65%, Tau^2^ = 0.03 *p* = 0.01]) (Fig. 2A). Sensitivity analysis on study type found a positive association in case controls (Unadjusted (five studies) OR 1.52 [95% CI 1.16; 1.98] *p* = 0.001, [I^2^ = 68%, Tau^2^ = 0.04 *p* = 0.01]; Adjusted OR 1.34 [95% CI 1.08; 1.65] *p* = 0.007, [I^2^ = 67.8%, Tau^2^ = 0.16 *p* = 0.01]). Unfortunately, we were not able to analyze cohort studies separately (only two studies available). This association remained significant after sensitivity analysis on study quality (Unadjusted OR 1.34 [95% CI 1.06; 1.70] *p* = 0.01, [I^2^ = 62%, Tau^2^ = 0.03 *p* = 0.03]; Adjusted OR 1.27 [95% CI 1.03; 1.57] *p* = 0.02, [I^2^ = 65%, Tau^2^ = 0.03 *p* = 0.01]).

Next, we did cross-stratification between each specific AID and ASD. We found a specific association between maternal type 1 diabetes (T1D) (Unadjusted (four studies) OR 1.80 [95% CI 1.32; 2.47] *p* = 0.0002, [I^2^ = 0%, Tau^2^ = 0 *p* = 0.98], adjusted analysis (three studies) OR 1.60 [95% CI 1.18; 2.18] *p* = 0.002 [I^2^ = 0%, Tau^2^ = 0 *p* = 0.85], psoriasis (Unadjusted (three studies) OR 1.36 [95% CI 1.01; 1.82] *p* = 0.04 [I^2^ = 0%, Tau^2^ = 0 *p* = 0.43], adjusted (three studies) OR 1.45 [95% CI 1.14; 1.85] *p* = 0.002, [I^2^ = 0%, Tau^2^ = 0 *p* = 0.7]), Inflammatory arthritis (IR) Unadjusted (four studies) OR 0.96 [95% CI 0.48; 1.90] *p* = 0.89, [I^2^ = 44%, Tau^2^ = 0.22 *p* = 0.15]; Adjusted (three studies), OR 1.38 [95% CI 1.14; 1.68] *p* = 0.001, [I^2^ = 0.8%, Tau^2^ = 0 *p* = 0.57], and increase risk of ASD in the offspring. In the contrary, we did not found any association between maternal inflammatory bowel diseases (IBD) (including Crohn disease and Ulcerative colitis), systemic lupus erythematosus (SLE) and child ASD (respectively, IBD: Unadjusted (five studies) OR 1.18 [95% CI 0.55; 2.54] *p* = 0.67, [I^2^ = 76%, Tau^2^ = 0.5 *p* < 0.01]; Adjusted (four studies) 1.03 [95% CI 0.72; 1.49] *p* = 0.85, [I^2^ = 58%, Tau^2^ = 0.07 *p* = 0.07] and SLE: Unadjusted (four studies) OR 1.03 [95% CI 0.72; 1.49] *p* = 0.15, [I^2^ = 0%, Tau^2^ = 0 *p* = 0.42]; only two adjusted studies). (Supplementary Fig. 2)

We also found a positive association between AID in fathers and ASD in the offspring (Unadjusted (six studies) OR 1.23 [95% CI 1.04; 1.44] *p* = 0.01, [I^2^ = 44%, Tau^2^ = 0 *p* = 0.11] (Supplementary Fig. 1B) adjusted OR (five studies) 1.18 [95% CI 1.07; 1.30] *p* = 0.01, [I^2^ = 15.5%, Tau^2^ = 0.002 *p* = 0.47]) (Fig. 2B). The same results were found after sensitivity analysis on quality (Unadjusted OR 1.24 [95% CI 1.04; 1.42] *p* = 0.01, [I^2^ = 53%, Tau^2^ = 0.007 *p* = 0.07] and adjusted 1.18 [95% CI 1.07; 1.30] *p* = 0.01, [I^2^ = 15.5%, Tau^2^ = 0.002 *p* = 0.47]) or type of study for which only case controls could be analyzed (Unadjusted OR 1.29 [95% CI 1.02; 1.63] *p* = 0.003, [I^2^ = 54%, Tau^2^ = 0.02 *p* = 0.06] and adjusted: 1.18 [95% CI 1.03; 1.35] *p* = 0.01, [I^2^ = 40%, Tau^2^ = 0.08 *p* = 0,17]).

In cross-stratification analysis, paternal T1D (Unadjusted (three studies) OR 1.79 [95% CI 0.88; 3.66] *p* = 0.1, [I^2^ = 42%, Tau^2^ = 0.16 *p* = 0.18, Adjusted (three studies) OR 1.42 [95% CI 1.10; 1.83] *p* = 0.007, [I^2^ = 0%, Tau^2^ = 0 *p* = 0.79) was associated with ASD in the offspring. Unlike mothers, there was no association between paternal psoriasis, IBD, and ASD (respectively, Psoriasis Unadjusted (three studies) OR 2.20 [95% CI = 1.17; 2.74] *p* = 0.0001, [I^2^ = 0%, Tau^2^ = 0 *p* = 0.66], Adjusted (four studies) OR 1.31 [95% CI 0.87; 1.99] *p* = 0.2, [I^2^ = 32.5%, Tau^2^ = 0.05 *p* = 0.30]; IBD Unadjusted (four studies) OR 1.26 [95% CI = 0.52; 3.04] *p* = 0.61, [I^2^ = 77%, Tau^2^ = 0.53 *p* < 0.01], Adjusted (four studies) OR 1.09 [95% CI 0.87; 1.37] *p* = 0.46, [I^2^ = 0%, Tau^2^ = 0 *p* = 0.15]) (Supplementary Fig. 3). There were not enough studies to study paternal LES and IR.

### ADHD in the offspring and AID in parents

**Fig. 3:**
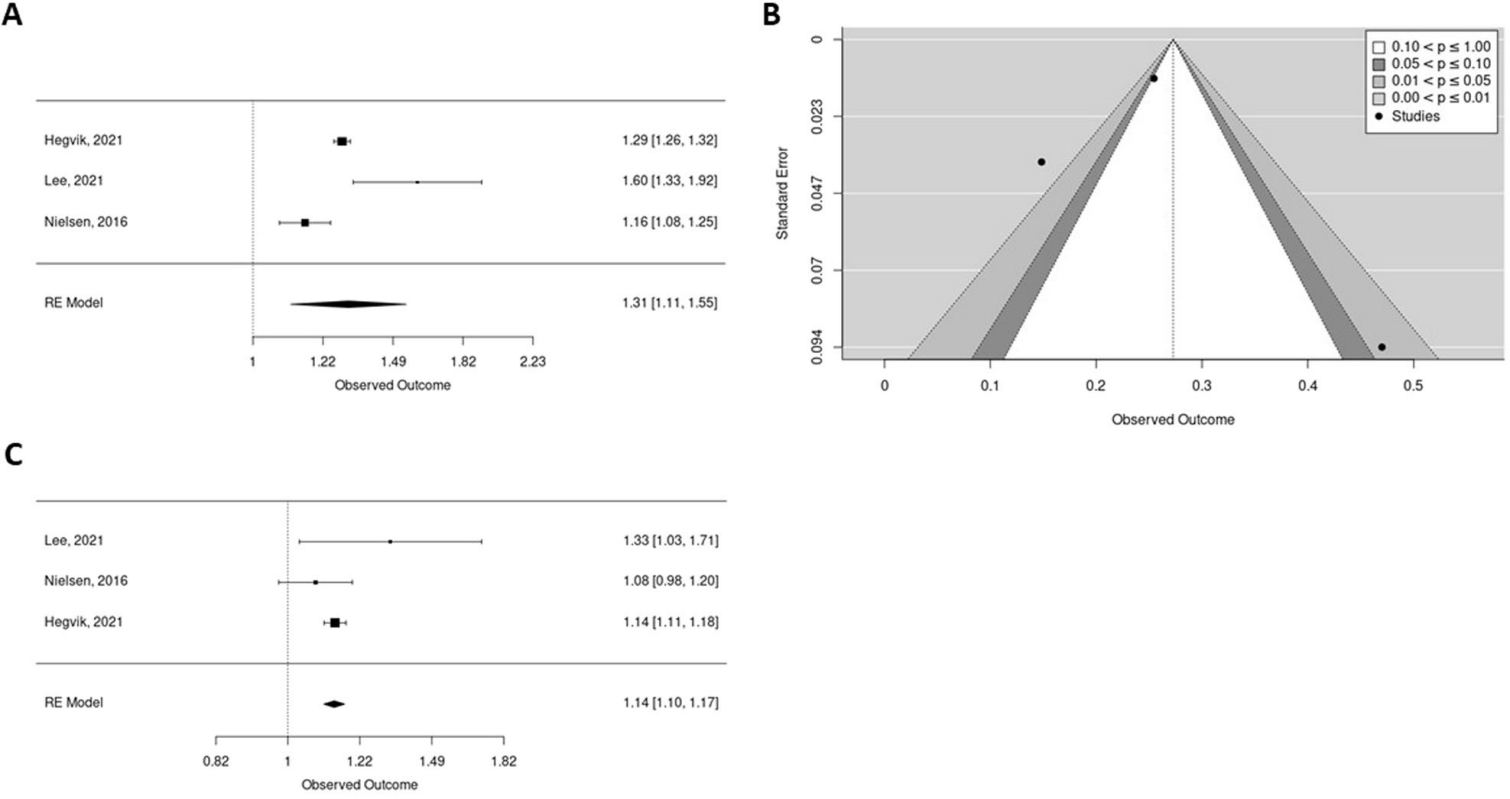
Forest plot and contour enhanced funnel plot showing the meta-analysis results of the association between ADHD in parents and ASD in the offspring (adjusted). **A** Mothers, **B** Fathers. Each square represents individual study effect. Its size represents the study weight in the overall analysis. The black lines on either side of the squares represent the confidence intervals. The diamond at the bottom represents the summary effect with the outer edges representing the confidence intervals. Square or diamond on the right of the central bar (i.e., superior to 1) represents a positive association between paternal AID and ASD in the offspring. To be significant, the confidence interval lines must not cross 1. **C** Contour enhanced funnel plot for mothers’ analysis. Areas represent studies with *p*-values larger than 0.10 (white), smaller than 0.05 (light gray), smaller than 0.01 (dark gray), and smaller than 0.001 (light gray outside large triangle).

Maternal AID were also associated with ADHD in the offspring (Unadjusted, only two studies, adjusted OR 1.31 [95% CI 1.11; 1.55] *p* = 0.001, [I^2^ = 93%, Tau^2^ = 0.13 *p* = 0.001]) (Fig. 3A). Heterogeneity was likely due to factors other than publication bias and no outlier was found (Fig. 3B). Sensitivity analysis on quality could not be performed (only two studies). Cross-stratification analysis found a positive association between maternal T1D (Unadjusted, only two studies, adjusted OR 1.36 [95% CI 1.24; 1.52] *p* < 0.0001, [I^2^ = 0%, Tau^2^ = 0 *p* = 0.82]), psoriasis (Unadjusted, only two studies, adjusted OR 1.41 [95% CI 1.29; 1.54] *p* < 0.0001, [I^2^ = 22%, Tau^2^ = 0.04 *p* = 0.31], IR (Unadjusted, only two studies, adjusted OR 1.32 [95% CI 1.25; 1.40] *p* < 0.0001, [I^2^ = 0%, Tau^2^ = 0 *p* = 0.78]). In the contrary, we did not found association in case of maternal IBD (Unadjusted, only two studies, adjusted OR 1.40 [95% CI 0.90; 1.40] *p* = 0.13, [I^2^ = 96%, Tau^2^ = 0.37 *p* = 0.003]) (Supplementary Fig. 4).

AID in fathers were associated with ADHD in offspring (Unadjusted, only two studies, adjusted OR 1.14 [95% CI 1.10; 1.17] *p* < 0.0001, [I^2^ = 0%, Tau^2^ = 0 *p* = 0.29]) (Fig. 3C). Results were similar in high quality studies only (adjusted OR 1.14 [95% CI 1.10; 1.17] *p* < 0.0001, [I^2^ = 0%, Tau^2^ = 0 *p* = 0.29). In cross-stratification we found a positive association between paternal T1D (Unadjusted, only two studies, adjusted OR 1.19 [95% CI 1.08; 1.31] *p* = 0.0003, [I^2^ = 0%, Tau^2^ = 0 *p* = 0.31]), psoriasis (Unadjusted, only two studies, adjusted OR 1.18 [95% CI 1.12; 1.24] *p* < 0.0001, [I^2^ = 0%, Tau^2^ = 0 *p* = 0.19]) but not IR (Unadjusted, only two studies, adjusted OR 1.28 [95% CI 0.89; 1.83] *p* = 0.18, [I^2^ = 78%, Tau^2^ = 0.07 *p* = 0.02]), IBD (Unadjusted, only two studies, adjusted OR 1.02 [95% CI 0.82; 1.27] *p* = 0.84, [I^2^ = 80%, Tau^2^ = 0.02 *p* = 0.037]) and ADHD in the offspring (Supplementary Fig. 5).

### Other NDD in the offspring and AID in parents

When pooling other NDD we found no association with AID in mothers (unadjusted OR 1.45 [95% CI 0.89; 2.37] *p* = 0.01, [I^2^ = 78%, Tau^2^ = 0.1 *p* = 0.01]. Heterogeneity was likely due to publication bias without outlier. This association remained identical in sensitivity analysis study type (no cohort study). Sensitivity analysis on quality score could not be performed (only two studies). Cross-stratification could not be performed due to the small number of studies.

No association were found in case of AID in fathers (Unadjusted OR 1.08 [95% CI 0.98; 1.19] *p* = 0.13, [I^2^ = 13%, Tau^2^ = 0.001 *p* = 0.32]. No sensitivity analysis could be performed.

## Discussion

Whereas previous meta-analyses have focused either on specific NDD/AID or on unspecific familial risk association [17, 18], our study is the first meta-analysis exploring separately the risk of maternal or paternal AID and offspring NDD.

First, we found that paternal and maternal AID conferred a significant risk factor for ASD and ADHD in the offspring. Several hypotheses may underlie these results: (i) environmental factors are highly implicated in the onset of AID. Among them, exposition to environmental pollutants (such as pesticides) or smoking are well recognized [43]. Environmental pollutants are also associated with increased risk of NDD [44, 45]. Thus, we cannot formally exclude that the association found between parental AID and NDD is not, at least partly, due to a common exposure to pollutants in connection with the same residential area. Both paternal and maternal smoking are also associated with an increased risk of NDD in the offspring [46, 47]. However, even if a direct effect of smoking on fetal brain exist, smoking in father may act through genetic pathways [48]. (ii) AID susceptibility genes might also act as risk factors for NDD. Genes involved in cytokines or in HLA system are associated with both immune functions and normal neurodevelopment (for review see ref. [49]). For example, in T1D, *class II HLA* genes—specifically *HLA-DR3/DR4* and *HLA DQ2/DQ8*—are known to be associated with its onset [50]. Interestingly, *HLA-DR4* is also associated with NDD with an estimated odds ratio of 4.67 [95% CI: 1.34–16.24] [51, 52]. Several studies have reported that HLA-DR proteins are expressed within several brain regions, such as the striatum, and participate in brain architecture [53]. This could explain in turn the gene-driven association between T1D and NDD [54, 55]. Despite the level of association between susceptibility genes in NDD and T1D remained weak, few studies have explored the association of T1D polygenic risk score on the risk of NDD [56–58]. Given that an increased risk of AID has been found in second and third degree relatives of patients with neurodegenerative disorders, shared environmental exposure is unlikely [41]. We therefore hypothesize that the shared risk of AID in mothers and fathers and NDD in offspring may be mediated by genetic pathways.

We also observed that AID in mothers appears to be a higher risk factor for NDD than in fathers (ASD 1.37 [95% CI: 1.16–1.61] versus 1.18 [95% CI: 1.03–1.44; ADHD 1.31 [95% CI: 1.11–1.55] versus 1.14 [95% CI: 1.10–1.31;]. Even if small overlap in CI is observed, the difference in OR between mothers and fathers is surprisingly stable (around 0.2). If AID in mother is an additional risk factor affecting fetal brain development, we hypothesize that it could act as an environmental insult. Maternal immune activation (MIA) induces by active AID during pregnancy could mediate this association, as seen in infection during pregnancy, known to increase risk of NDD in the offspring. According to animal models of MIA, dysregulation of specific immunological pathways during pregnancy is associated with NDD in children [59]. In MIA-mice model, interleukin 6 (IL-6) and interleukin 17 (IL-17) secretion during pregnancy mediates the occurrence of ASD-like behavior in pups [60, 61]. The mediation from MIA of gestational mothers to ASD-like behaviors in pups remained unclear but preliminary studies suggested the action of IL-17 on specific receptors, located on the fetal neurons [60]. Thus, injection of anti IL-17 antibody into pregnant MIA-mice model, reduces the development of the ASD-like phenotypes in the pups [60].

Our study should be considered in light of its general strengths and limitations, mostly related to the meta-analysis method. One of the major strengths of our report is its ability to gather a large number of studies encompassing several millions of individuals, warranting the robustness and the reliability of the results we reported. In the same line, our main results were calculated as adjusted OR which considered the effect due to confounding variables and allowed the generalization of our findings. Third, we have only included studies including both mothers and fathers in order to control the different measured confounder such as diagnostic criteria for both NDD and AID. By contrast, the intrinsic conception of the studies we included in the meta-analysis has several weaknesses. First, there was relatively less data on fathers making any definitive conclusion difficult. This problem is particularly acute when we consider cross-stratification analyses. Second, our study was not exhaustive in its ability to consider the whole group of NDD (for example no study have focused on IA or specific learning disorders) in offspring and/or AID in parents, even if we considered the main NDD and AID in our analysis. Despite all, we hypothesize that the association between AID and NDD in offspring can be considered as the main rule even though some exceptions (such as IBD) may exist for other rarer conditions that we could not take into account in our study. We have also consciously chosen to include, for analysis purposes, only diagnosed NDD, excluding de facto subclinical symptoms. However, environmental risk factors increase subclinical neurodevelopmental symptoms, suggesting that we may underestimate the impact of parent AID on NDD. As autoimmune diseases are rare in male, and in order to overcome these limitations, we advocate the development of international prospective cohort studies including fathers and mothers with several AID in order to assess the role of genetics in this association (or not) with NDD. Third, we only have little information on parents’ AID. If we consider that AID in mother could be an extra risk factor mediated by environment, we do not know whether AID was clinically active during the specific period of pregnancy. However, the absence of clinical symptoms does not mean the absence, for example, of low-grade subsyndromic inflammation. Finally, in most of the reports considered for the meta-analysis, the potential use of treatments by mothers during pregnancy was not reported. Most drugs currently prescribed in AID have a pleiotropic effect and could participate in the increased risk of NDD in the offspring of mothers with AID [62]. However, the follow-up of children exposed to immunosuppressive drugs during pregnancy did not argue for this hypothesis [63–65]. Prospective studies including mothers with an AID onset before, during and after the pregnancy and screening both the clinical, treatments and biological status would fill this gap and help to disentangle the genetic and environmental involvement.

In conclusion, our findings help to reconsider the relationship between AID in parents and the NDD risk in offspring. Our results point to a complex mechanism combining common factors in fathers and mothers (such as genetics) accounting for half of the risk and a specific maternal factor, possibly mediated by the direct effect of MIA on fetal neurodevelopment, accounting for the other half. Future studies considering both genetic and environmental information may be of great value to help deciphering the intriguing link between AID in parents and NDD in the offspring. Although the effect size remains modest, more systematic screening for NDD in children born to parents with AID should be considered.

## Supplementary information

Supplementary material

## Data Availability

Data used are available on reasonable request.

## Materials availability

Other materials used are available on reasonable request

## Code availability

Code used is available on reasonable request.
