# Supplementary material for: Parental autoimmune and autoinflammatory disorders as multiple risk factors for common neurodevelopmental disorders in offspring: a systematic review and meta-analysis
Source: Transl Psychiatry. 2022 Mar 18;12:112. doi: 10.1038/s41398-022-01843-y (PMC8933391; doi:10.1038/s41398-022-01843-y)
Supplement: Supplementary file 1 — Supplementary material [file 41398_2022_1843_MOESM1_ESM.docx]

**Search strategy**

([Intellectual Disabilities] OR [Intellectual Developmental Disorder] OR [mental retardation] OR [Global Developmental Delay] OR [Communication Disorders] OR [Language Disorder] OR [Speech Sound Disorder] OR [Childhood-Onset Fluency Disorder] OR [Social Communication Disorder] OR [Pragmatic Communication Disorder] OR [Autism spectrum disorder] OR [Autism] OR [Asperger syndrome] OR [Asperger] OR [ASD] OR [autistic disorder] OR [attention deficit hyperactivity disorder] OR [adhd] OR [Attention Deficit Disorder with Hyperactivity] OR [Hyperkinetic Disorder] OR [Hyperactivity disorder] OR [hyperactive child syndrome] OR [Attention Deficit Disorder] OR [ADD] OR [attention deficit] OR [minimal brain dysfunction] OR [Specific Learning Disorder] OR [Developmental Coordination Disorder] OR [Stereotypic Movement Disorder] OR [Tic] OR [Tic Disorders] OR [Tourette's disorders] OR [Neurodevelopmental Disorder]) AND ([maternal] OR [mother] OR [father] OR [paternal] OR [parental] OR [first degree relative] OR [parent]) AND ([inflammatory diseases] OR inflammatory disorders] OR [Behçet disease] OR [Blau’s syndrome] OR [Early onset sarcoidosis] OR [Cryopyrin associated periodic syndrome] OR [Chronic recurrent multifocal osteomyelitis] OR [Deficiency of IL-1 receptor antagonist] OR [Familial Mediterrean Fever] OR [Mevalonate kinase deficency] OR [Hyper IgD syndrome] OR [NLRP12 -associated periodic syndrome] OR [Pyogenic Sterile Arthritis] OR [Pyoderma Gangrenosum and Acne syndrome] OR [PAPA] OR [Tumor necrosis factor receptor-associated periodic syndrome] OR [TRAPS] OR Periodic fever, aphthous stomatitis, pharyngitis and cervical adenitis] OR [PFAPA] OR [marshall syndrome] OR [CANDLE syndrome] OR [DITRA syndrome] OR [Schnitzler syndrome] OR [di Majeed syndrome] OR [Undefined Periodic fever] OR [autoimmune diseases] OR [autoimmune disorders] OR [Addison’s disease] [Adult Still's disease] OR [Agammaglobulinemia] OR [Alopecia areata] OR [Amyloidosis] OR [Ankylosing spondylitis] OR [Anti-GBM/Anti-TBM nephritis] OR [Antiphospholipid syndrome] OR [Autoimmune angioedema] OR [Autoimmune dysautonomia] OR [Autoimmune encephalomyelitis] OR [Autoimmune hepatitis] OR [Autoimmune inner ear disease] OR [AIED] OR [Autoimmune myocarditis] OR [Autoimmune oophoritis] OR [Autoimmune orchitis] OR [Autoimmune pancreatitis] OR [Autoimmune retinopathy] OR [Autoimmune urticaria] OR [Axonal neuronal neuropathy] OR [AMAN] OR [Baló disease] OR [Behcet’s disease] OR [Benign mucosal pemphigoid] OR [Bullous pemphigoid] OR [Castleman disease] OR [Celiac disease] OR [Chagas disease] OR [Chronic inflammatory demyelinating polyneuropathy] OR [CIDP] OR [Chronic recurrent multifocal osteomyelitis] OR [CRMO] OR [Churg-Strauss Syndrome] OR [Eosinophilic Granulomatosis] OR [EGPA] OR [Cicatricial pemphigoid] OR [Cogan’s syndrome] OR [Cold agglutinin disease] OR [Congenital heart block] OR [Coxsackie myocarditis] OR [CREST syndrome] OR [Crohn’s disease] OR [Dermatitis herpetiformis] OR [Dermatomyositis] OR [Devic’s disease] OR [neuromyelitis optica] OR [Discoid lupus] OR [Dressler’s syndrome] OR [Endometriosis] OR [Eosinophilic esophagitis] OR [Eosinophilic fasciitis] OR [Erythema nodosum] OR [Essential mixed cryoglobulinemia] OR [Evans syndrome] OR [Fibromyalgia] OR [Fibrosing alveolitis] OR [Giant cell arteritis] OR [temporal arteritis] OR [Giant cell myocarditis] OR [Goodpasture’s syndrome] OR [Granulomatosis with Polyangiitis] OR [Graves’ disease] OR [Guillain-Barre syndrome] OR [Hashimoto’s thyroiditis] OR [Hemolytic anemia]OR [Henoch-Schonlein purpura] OR [Herpes gestationis] OR [pemphigoid gestationis] OR [Hidradenitis Suppurativa] OR [Hypogammalglobulinemia] OR [IgA Nephropathy] OR [IgG4-related sclerosing disease] OR [Immune thrombocytopenic purpura] OR [Inclusion body myositis] OR [Interstitial cystitis] OR [Juvenile arthritis] OR [Juvenile diabetes] OR [Type 1 diabetes] OR [Gestational diabetes] OR [Juvenile myositis] OR [Kawasaki disease] OR [Lambert-Eaton syndrome] OR [Leukocytoclastic vasculitis] OR [Lichen planus] OR [Lichen sclerosus] OR [Ligneous conjunctivitis] OR [Linear IgA disease] OR [Lupus] OR [Lyme disease chronic] OR [Meniere’s disease] OR [Microscopic polyangiitis] OR [Mixed connective tissue disease] OR [Mooren’s ulcer] OR [Mucha-Habermann disease] OR [Multifocal Motor Neuropathy] OR [Multiple sclerosis] OR [Myasthenia gravis] OR [Myositis] OR [Narcolepsy] OR [Neuromyelitis optica] OR [Ocular cicatricial pemphigoid] OR [Optic neuritis] OR [Palindromic rheumatism] OR [PANDAS] OR [Paraneoplastic cerebellar degeneration] OR [Paroxysmal nocturnal hemoglobinuria] OR [Parry Romberg syndrome] OR [Pars planitis] OR [Parsonage-Turner syndrome] OR [Pemphigus] OR [Perivenous encephalomyelitis] OR [Pernicious anemia] OR [POEMS syndrome] OR [Polyarteritis nodosa] OR [Polyglandular syndromes] OR [Polymyalgia rheumatica] OR [Polymyositis] OR [Primary biliary cirrhosis] OR [Primary sclerosing cholangitis] OR [Progesterone dermatitis] OR [Psoriasis]OR [Psoriatic arthritis] OR [Pure red cell aplasia] OR [Pyoderma gangrenosum] OR [Raynaud’s phenomenon] OR [Reflex sympathetic dystrophy]OR [Relapsing polychondritis] OR [Restless legs syndrome] OR [Retroperitoneal fibrosis] OR [Rheumatic fever] OR [Rheumatoid arthritis] OR [Sarcoidosis] OR [Schmidt syndrome] OR [Scleritis] OR [Scleroderma] OR [Sjögren’s syndrome] OR [Sperm and testicular autoimmunity] OR [Stiff person syndrome] OR [Susac’s syndrome] OR [Sympathetic ophthalmia] OR [Takayasu’s arteritis] OR [Temporal arteritis] OR [Giant cell arteritis] OR [Thrombocytopenic purpura] OR [Tolosa-Hunt syndrome] OR [Transverse myelitis] OR [Ulcerative colitis] OR [Undifferentiated connective tissue disease] OR [Uveitis] OR [Vasculitis] OR [Vitiligo] OR [Vogt-Koyanagi-Harada Disease])

**Deviations from the initial protocol**

We did not find any studies that looked separately at AID in fathers. As studies of mothers only were much more numerous and could bias the results, we decided to include only studies of fathers and mothers. In the same vein, we decided to exclude gestational diabetes from the analyses, because (i) it can obviously only be found in mothers (ii) its mediation by immunity is not certain.


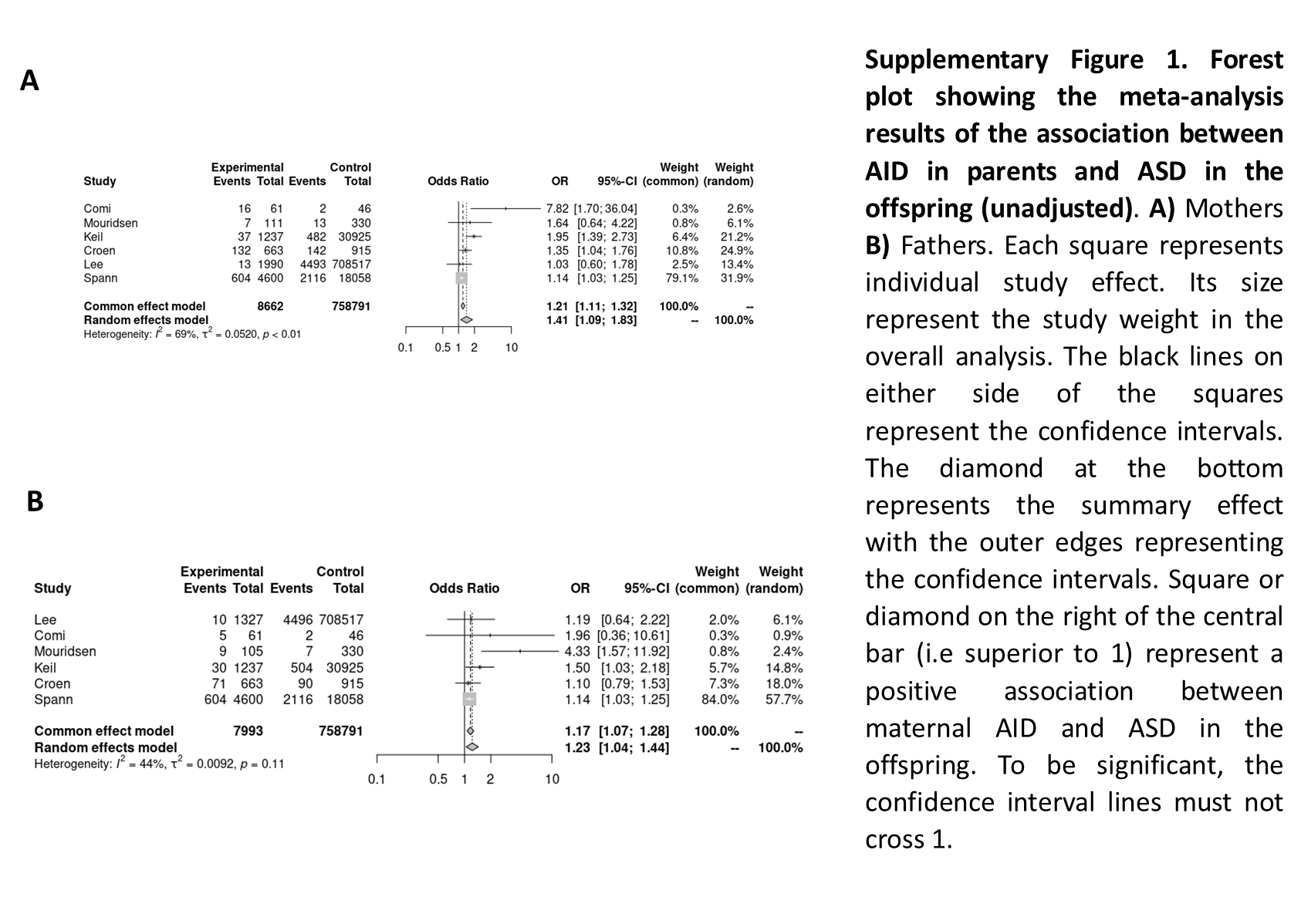


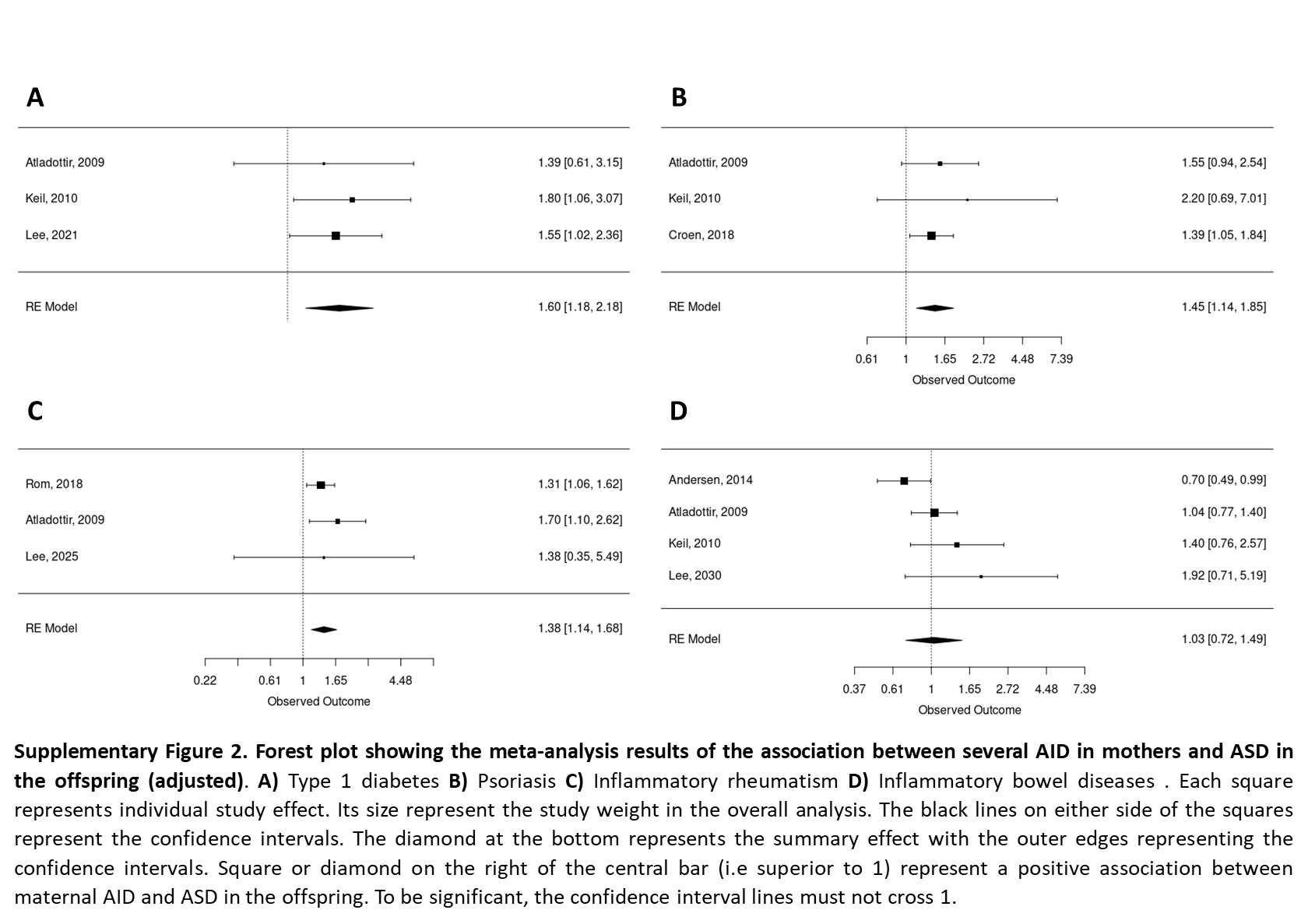

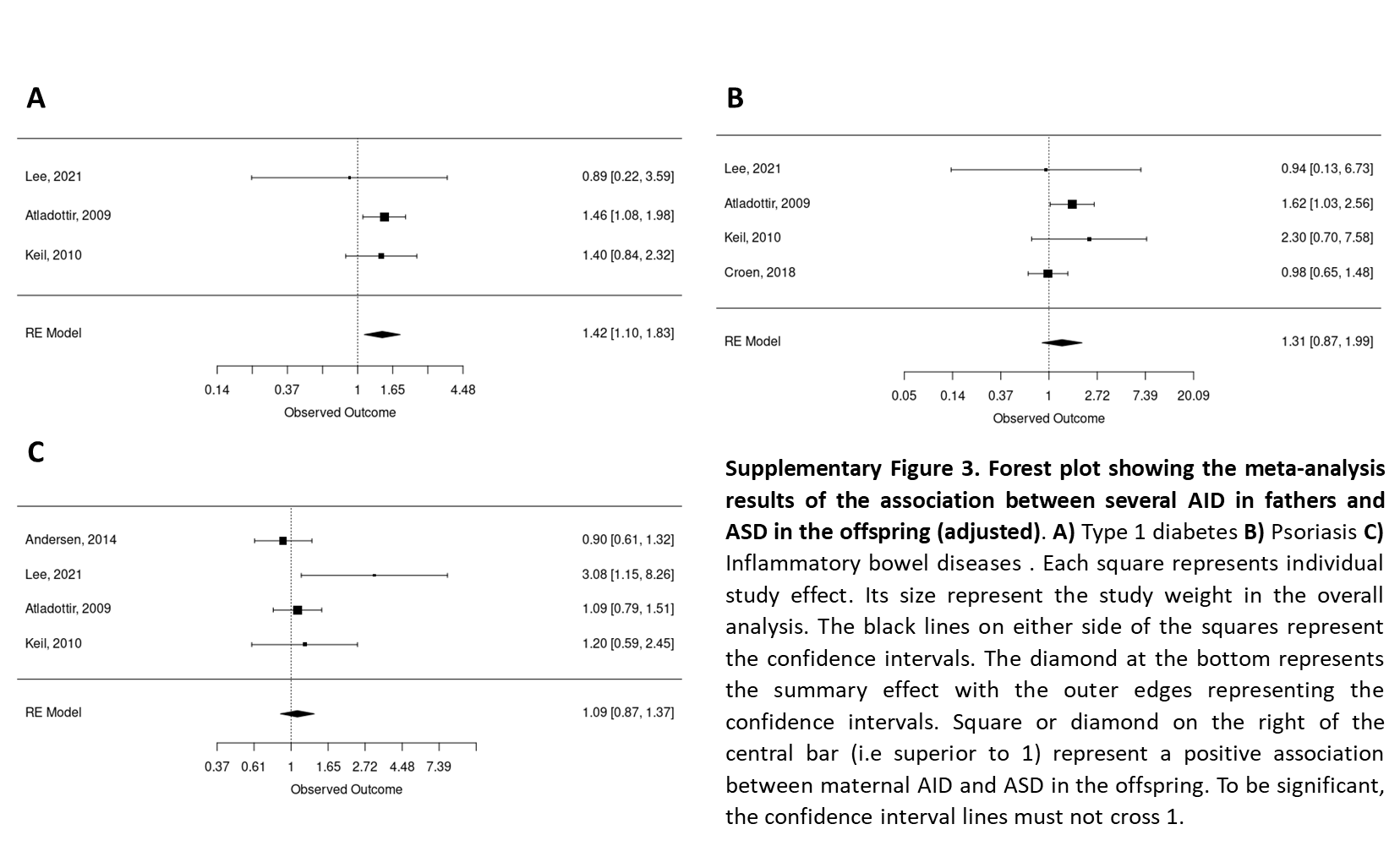


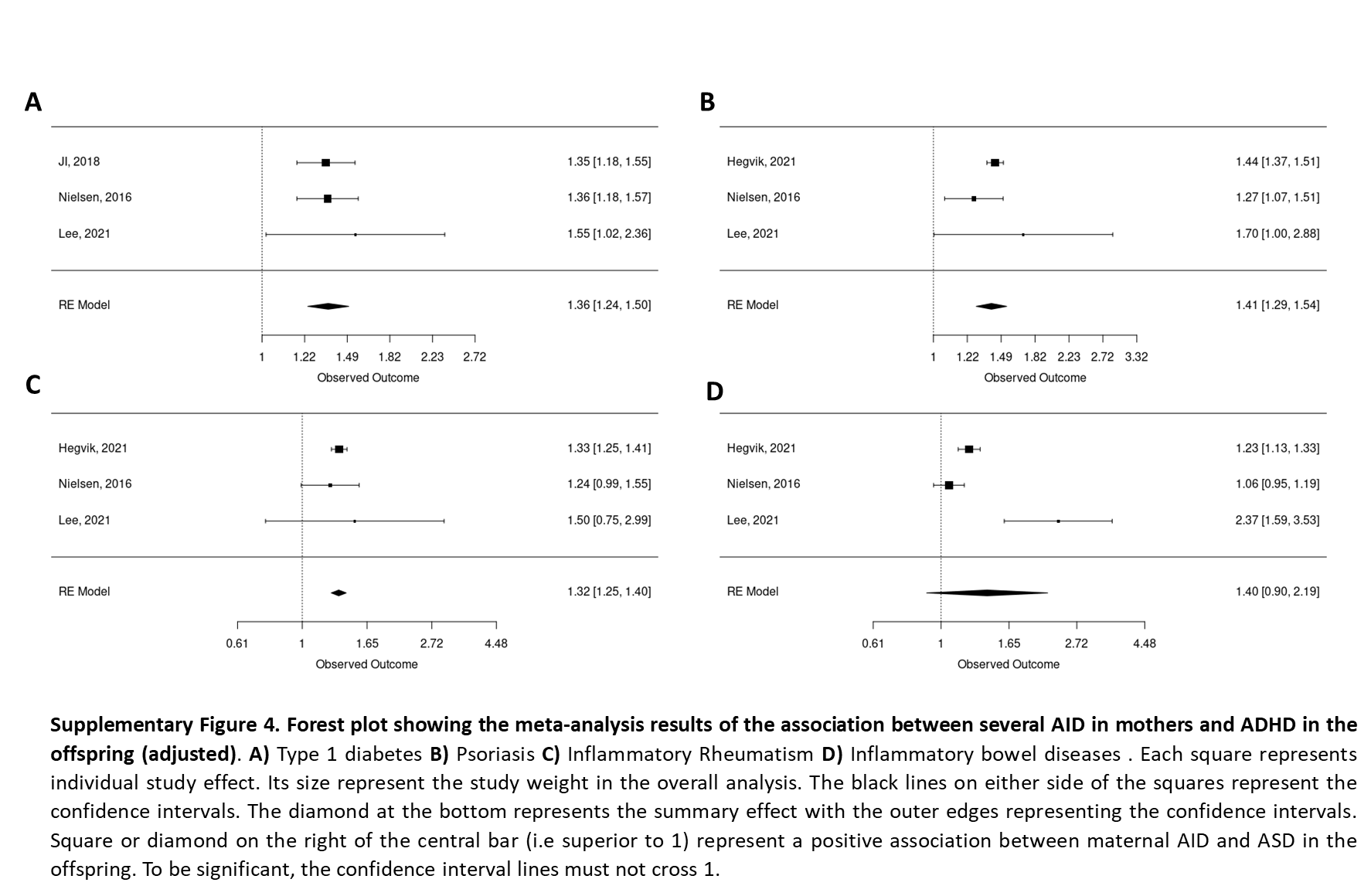


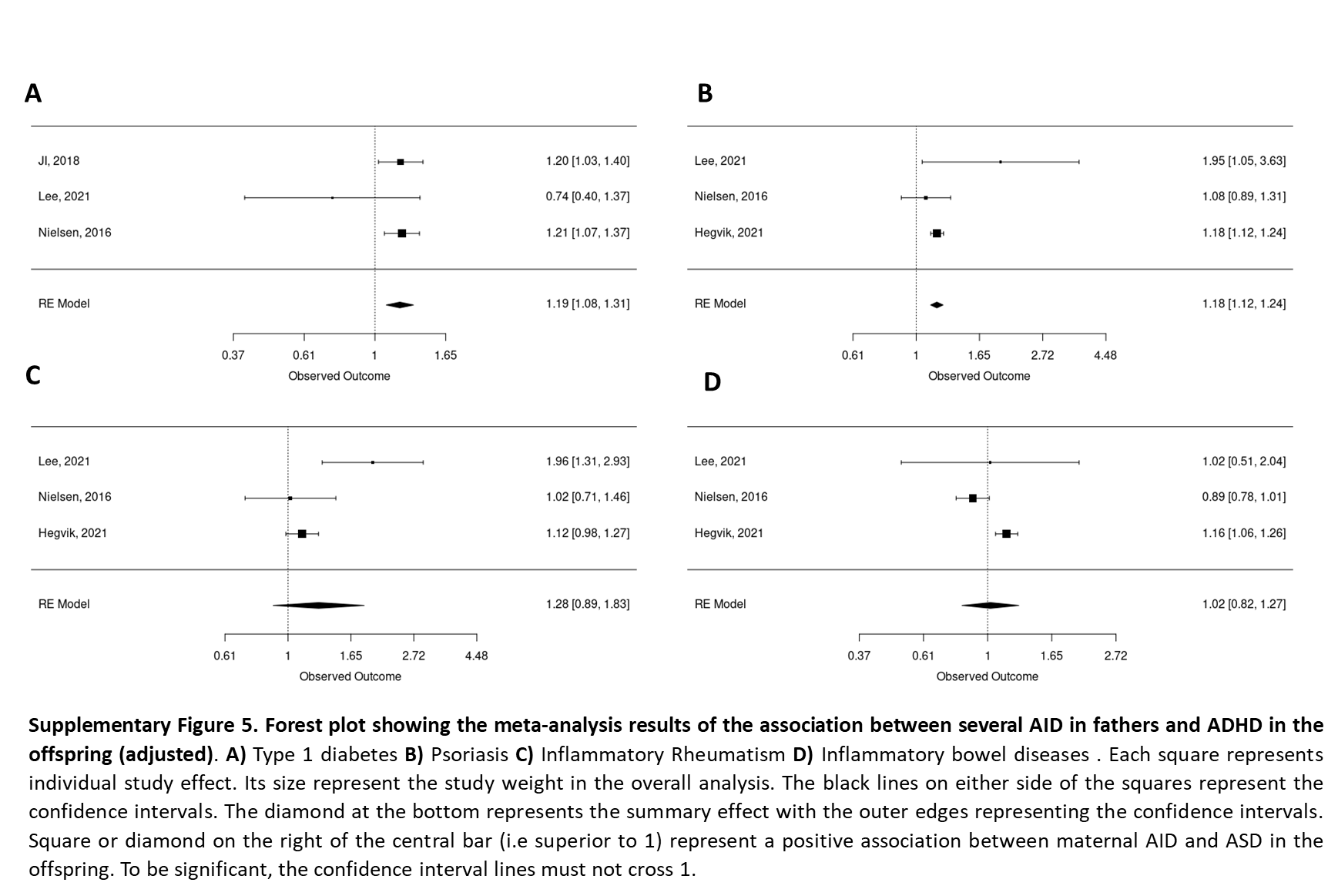

**Supplementary table 1. Descriptive data for cohort studies included in the meta-analysis.** AID: autoimmune or inflammatory disorders; NDD: Neurodevelopmental disorders; T1D Type 1 Diabetes, RA: Rheumatoid arthritis; IBD: Inflammatory bowel disease; ADHD: Attention deficit/Hyperactivity disorders; ASD: Autism spectrum disorders; (f): fathers, (m): mothers. Note that for Lee, we have deliberately shown two different lines because they studied two neurodevelopmental outcomes.

**Supplementary table 2. Descriptive data for cross-sectional studies included in the meta-analysis.** AID: autoimmune or inflammatory disorders; NDD: Neurodevelopmental disorders; T1D Type 1 Diabetes, RA: Rheumatoid arthritis; IBD: Inflammatory bowel disease; UC: Ulcerative colitis; MS: Multiple sclerosis; SLE: Systemic Lupus erythematosus; RA: Rheumatoid arthritis ; JA: Juvenile arthritis; AS: Ankylosing spondylitis ADHD: Attention deficit/Hyperactivity disorders; ASD: Autism spectrum disorders; PDD: Pervasive developmental disorder; IA: Infantile autism; TS: Tourette syndrome; DD: developmental disorders; (f): fathers, (m): mothers. Note that for the studies of Altadottir and Croen, we have deliberately shown two different lines because they each studied two neurodevelopmental outcomes.
